# Evaluation of Intimate Partner Violence and Its Association With Depression Among Women in Chengalpattu District, India: A Cross-Sectional Study

**DOI:** 10.7759/cureus.59825

**Published:** 2024-05-07

**Authors:** Rathibala Arumugaperumal, Gowthamkarthic Ravichandhiran, Soumya Agadi, Vigneshsamy Muthuchamy, Raja D, Suganthi S, Jasmine Maruthupandian

**Affiliations:** 1 Department of Community Medicine, Chettinad Hospital and Research Institute, Chettinad Academy of Research and Education, Kelambakkam, IND; 2 Department of Community Medicine, KLE Jagadguru Gangadhar Mahaswamigalu Moorsavirmath Medical College (JGMMMC), KLE Academy of Higher Education and Research, Hubballi, IND; 3 Department of Community Medicine, PSP Medical College Hospital and Research Institute, Oragadam, IND; 4 Department of Community Medicine, Shri Sathya Sai Medical College and Research Institute, Ammapettai, IND

**Keywords:** who (violence against women instrument), phq-9, depression, spouse abuse, domestic violence, intimate partner violence

## Abstract

Background

Intimate partner violence is one of the major problems experienced by women all over the world. Almost one in three women have experienced one or more forms of violence at least once in their lifetime. These estimates confirm that physical and sexual intimate partner violence remains pervasive in the lives of women across the globe. Intimate partner violence has been found to have a severe impact on mental health such as depression symptoms. This study aimed to assess the prevalence of intimate partner violence in terms of controlling behavior, sexual, and physical violence, and its association with depression in women of Chengalpattu district, India.

Methods

It is a community-based cross-sectional study conducted in 12 villages under the field practicing area of the rural health training center of Chettinad Health and Research Institute in Kelambakkam, India, by simple random sampling among 190 women participants ever married or partnered of age ≥18 years. A pre-tested, semi-structured questionnaire was used which included the WHO Violence Against Women Instrument (VAWI) in the English language and Patient Health Questionnaire 9 (PHQ 9). The collected data was entered in Microsoft Office Excel (Microsoft Corporation, Redmond, United States) and analyzed using IBM SPSS Statistics for Windows, Version 21 (Released 2012; IBM Corp., Armonk, New York, United States). A probability value (p-value) of less than 0.05 was considered statistically significant.

Results

The mean (SD) age of the study participants was 34 (±8) years. Among the 190 participants, 57.4% reported controlling behavior, 31.1% reported physical violence and 7.4% reported sexual violence by the intimate partner at least once in the past 12 months. About 34.7% were found to have mild depression and 21.6% with moderate depression. There was a significant association between intimate partner violence and depression in women (p < 0.001)

Conclusion

The present study found that there is a huge impact of intimate partner violence on the mental health of women whether it is psychological, physical, or sexual. There is a need for awareness and effective management of violence against women, especially in rural areas. Strategies focusing on women's education, leadership, empowerment, decision-making, and financial independence are very much needed.

## Introduction

WHO defines intimate partner violence against women as any behavior by a current or former male intimate partner within the context of marriage, cohabitation, or any other formal or informal union, that causes physical, sexual, or psychological harm. On average, 736 million and up to 852 million women who were aged 15 years or older in 2018 (almost one in three women) have experienced one or more forms of violence at least once in their lifetime [1]. These estimates confirm that physical and sexual intimate partner violence remains pervasive in the lives of women across the globe [1].

Many married women don’t consider forced sex against their will as sexual violence because they are made to think it’s their duty to serve the husband; also, not all countries have legal laws for marital rape. About 10-15% of global sexual violence is present [1]. The importance of intimate partner violence is acknowledged in the fifth Sustainable Development Goal: “Eliminate all forms of violence against all women and girls in the public and private spheres.” It is also a risk factor for premature morbidity and mortality [2]. Women experience this violence more commonly than men. Female deaths due to male partners are shown to be one of the common types of murder of women [2].

According to National Family Health Survey 5 (NFHS-5), the percentage of ever-married women aged 18-49 years who have experienced spousal violence is 29.3% in India, and in Tamilnadu, the percentage is 38.1% and specifically in rural areas the percentage is 42.2%. Intimate partner violence not only affects the victim but also the families and society. For victims, apart from physical injuries, mental health problems especially depression and anxiety are common. More importantly, intimate partner violence is often repetitive and may increase in frequency and severity over time. The damage caused by different types of violence and multiple exposures may have an added impact, with serious impacts on mental health in the long term [3]. Cultural, social, and economic factors at the societal level, can create a context for violence if a couple couldn’t meet up the level of expectation [4].

Exposure to violence can exacerbate mental health conditions or can lead to psychopathological conditions, at the same time existing mental health issues can increase predisposition to intimate partner violence [5]. Screening for intimate partner violence in the healthcare setting is important and when identified if they are referred to a support system, these acts of violence could be reduced [6]. Frustration, stress, and lack of control can increase psychopathological problems that are associated with intimate partner violence which would further precipitate violent episodes [7]. Inequality in gender is one of the major contributors to depression [8,9]. Focused strategies are required for women's education, leadership, empowerment, decision-making, and financial independence [10].

This study aimed to assess the prevalence of intimate partner violence in terms of controlling behavior, sexual, and physical violence, and their association with depression in women of rural areas of Chengalpattu district, India.

## Materials and methods

A community-based cross-sectional study was conducted in 12 villages in the field practicing area of the Rural Health Training Centre (RHTC) of Chettinad Hospital and Research Institute in Kelambakkam, India. Inclusion criteria included, adult women who gave consent to the study, women residing in rural areas of Chengalpattu district, India, and women ever married or partnered. The exclusion criteria included those who were not able to communicate and were not willing to give consent. The period of study was for two months (June 2023 to August 2023).

The sample size was calculated based on the study conducted by Daruwalla et al. [2] where the prevalence of physical domestic violence among women was p = 13%. Using the formula n = z^2^pq/d^2^ (n is sample size, z = 1.96 standard normal distribution for reflecting the confidence interval, with allowable error (d) as 5), the sample size is 173, and adding the nonresponse rate of 10%, the final sample size is 190.

Ethical clearance was obtained from the Chettinad Academy of Research and Education Institutional Human Ethics Committee (CARE IHEC-II) IHEC-I/2001/23. Informed written consent was taken from the study participants.

Out of the 12 villages, 9 villages under the Thirukalkundram block includes, Mamallapuram, Kokilamedu, Poonjeri, Kadambadi, Manamai, Kunnathur, Nallur, Perumaleri, Kuzhipanthandalam. Three villages under the Thiruporur block include Karanai, Kunnapattu, and Paiyanoor as per the Indian census conducted in 2011. From these villages, 190 women of age ≥18 years were selected by probability proportional to size sampling. Each participant from each village was selected by simple random sampling.

Data was collected using a pre-tested semi-structured questionnaire. Modified BG Prasad scale updated for the year 2023 [11], WHO Violence Against Women Instrument (WHO VAWI) in the English language [12], and Patient Health Questionnaire 9 (PHQ-9) [13] for depression was used, WHO VAWI consists of 13 items that specifically evaluate psychological, physical and sexual violence to provide a comprehensive understanding of the prevalence and impact of violence against women. PHQ-9 consists of nine questions that evaluate respondents' rate of the frequency of specific symptoms over the past two weeks using a scale from 0 (not at all) to 3 (nearly every day). A score of 0-4 is minimal depression, 5-9 is mild depression, 10-14 is moderate depression, and 15 to 27 is severe depression. Minimal depression needs no intervention and other types require treatment.

Data was entered into a Microsoft Excel sheet (Microsoft Corporation, Redmond, United States) and analyzed by IBM SPSS Statistics for Windows, Version 21 (Released 2012; IBM Corp., Armonk, New York, United States). Categorical data were expressed in the form of percentages. Illustrations of data were done by using tables. The descriptive representation of data was done in the form of frequencies and percentages. A chi-square test was used to find an association between depression and risk factors. A probability value (p-value) of less than 0.05 was considered statistically significant. The strength of association is expressed as an odds ratio.

## Results

The mean age of 190 participants was 34±8 years. Among the participants, 34.7% were in the age group of 18 to 29 years, 31.1% were educated up to secondary school, 37.4% were unemployed, and most participants (49.5%) belonged to upper middle class (class II) socioeconomic status according to the modified BG Prasad classification (Table 1).

**Table 1:** Socio-demographic characteristics of participants (N = 190)

Variables		Frequency (N)	Percentage (%)
Age (in years)	18-29	66	34.7
	30-39	69	36.3
	40-49	53	27.9
	50-69	2	1.1
Education	Elementary School	41	21.6
	Secondary School	59	31.1
	Higher Secondary	36	18.9
	Diploma	21	11.1
	Undergraduate	27	14.2
	Postgraduate	6	3.2
Occupation	Unemployed	71	37.4
	Unskilled	65	34.2
	Semi-skilled	30	15.8
	Skilled	9	4.7
	Semi-professional	12	6.3
	Professional	3	1.6
Partner Occupation	Unemployed	3	1.6
	Unskilled	56	29.5
	Semi-skilled	87	45.8
	Skilled	38	20.0
	Semi-professional	6	3.2
Socio-economic status (modified BG Prasad Scale)	CLASS I	48	25.3
	CLASS II	94	49.5
	CLASS III	42	22.1
	CLASS IV	6	3.2

Among the 190 participants, 57.4% reported controlling behavior, 31.1% reported physical violence and 7.4% reported sexual violence by the intimate partner at least once in the past 12 months (Table 2).

**Table 2:** Types of intimate partner violence experienced by the participants (N = 190)

Type of violence	Frequency (N)	Percentage (%)
Controlling behavior	109	57.4
Physical	59	31.1
Sexual	14	7.4

About 42.1% had minimal depression and 34.7% had mild depression among the participants which was assessed by the PHQ 9 questionnaire. Depending on the severity of depression, those with mild, moderate, and severe depression were considered to have depression and those with minimal form were considered as not depressed, and the following association was done (Table 3).

**Table 3:** Frequency of depression among the participants (N = 190)

Level of depression	Frequency (N)	Percentage (%)
Minimal	80	42.1
Mild	66	34.7
Moderate	41	21.6
Severe	3	1.6

Significant associations (p < 0.05) were found between depressive symptoms and controlling behavior, as well as between physical and sexual violence and depression (Table 4).

**Table 4:** Association between interpersonal violence and depression p-value < 0.05 is significant.

Interpersonal violence		Depression		p-value	chi-square value
		Present	Absent		
Controlling behavior	Present	104	5	0.001	147.7
	Absent	6	75		
Physical violence	Present	59	0	0.001	55.1
	Absent	51	80		
Sexual violence	Present	14	0	0.001	7.99
	Absent	96	80		

## Discussion

The goal of this study was to assess the prevalence of intimate partner violence and its association with depression. In this discussion we focus on, analysis done on responses to questions about intimate partner violence.

Violence experienced by the participants

Among the study participants, 57.4% reported controlling behavior, 31.1% reported physical violence, and 7.4% reported sexual violence by their intimate partner at least once in the past 12 months. In the study by Daruwalla et al., the prevalence of physical domestic violence was 13%, sexual violence was 4%, and emotional violence was 19% [2]. In our study, controlling behavior and physical violence overlapped to a large extent similar to Lövestad et al. [14]. Similar to our study, Kapiga et al. also reported controlling behavior was the most prevalent type of abuse with 82% prevalence in lifetime suicidal behaviors and 63% during the past 12 months [15]. In the Australian Pregnancy cohort study, emotional intimate partner violence was found to be the common type [16]. The prevalence of domestic violence was found to be 54% in a study conducted in Kolkata [17]. About 19.8% had experienced psychological violence, and 18.6% had experienced both physical and psychological violence in their lifetime. Slapping, beating, and hitting were the commonest forms of physical violence, whereas insulting and threatening were the most common forms of psychological violence [18]. Sexual violence in this study is similar to the study done in the urban locality of Pune which shows sexual violence of 8.1% [18,19]. About 55.83% prevalence of domestic violence was seen in the Latur study [20]. According to a study done in Thailand, the prevalence of psychological violence was 41.2%, physical violence was 4.3%, and sexual violence was 2.3%. Several participants had experienced more than one form of violence [21].

Impact on mental health

According to the Diagnostic and Statistical Manual of Mental Disorders (DSM 5), people who suffer from depression have persistent feelings of sadness and hopelessness and lose interest in activities once enjoyed by them. Apart from the emotional problems caused by depression, individuals can also have physical symptoms such as chronic pain or digestive issues. To be diagnosed with depression, the symptoms must persist for at least two weeks. Experiencing five or more symptoms during the same two-week period and at least one of the symptoms should be either depressed mood or loss of interest/pleasure is the criterion to make a diagnosis of depression [22].

Mental health implications of violence include the risk of experiencing post-traumatic stress disorder, anxiety suicidal behaviors, depression, and substance use [22].

In this study, 42.1% had minimal depression, 34.7% had mild depression, 21.6% had moderate depression and 1.6% had severe depression which was assessed by the PHQ 9 questionnaire. Women experiencing controlling behavior had higher odds of depression than those experiencing physical and sexual violence which is similar to the findings from the study by Lövestad et al. where associations showed that women exposed to controlling behavior had higher odds of depression [14]. Women experiencing violence had higher levels of depression and post-traumatic stress disorder [23,24]. Depression was found to be an important consequence in eight studies and emotional violence was more associated with depression than sexual and physical violence [25]. According to Joshi et al., physical violence and controlling behavior by the husband were significantly associated with psychological abuse as also in Mishra et al. [26,27]. In this study, there was a significant association between intimate partner violence and depression (p < 0.001) similar to Kamimura et al., where participants experiencing intimate partner violence reported poor mental health (p < 0.01) and low self-esteem (p < 0.01) compared to those who didn’t go through intimate partner violence [28]. Sharma et al. study found that women who had experienced physical violence had significantly unhealthy mental status with an odds ratio of 2.52 and those who experienced sexual violence had unhealthy mental status with an odds ratio of 3.11 [29]. Experiencing both physical violence and controlling behavior concurrently was associated with a little larger-than-expected effect on mental health than the additive effect from individual forms of violence [30].

Strength of the study

This study was done using validated questionnaires like the WHO Violence Against Women Instrument (WHO VAWI) and PHQ-9. A probability sampling method was used which ensured that each unit had an equal chance of getting selected for the study. Not many studies have focused on rural women when it comes to intimate partner violence.

Limitation

The limitation of this study is that it did not assess the factors that contribute to intimate partner violence. This study was done in a small demographic area and hence cannot be generalized to a population with different demographic traits. Recall and recency biases might have played a role in the results.

Recommendations

Economic intervention like financial support for women's education, job opportunities, and financial freedom would reduce controlling behavior, and improve relationship quality and economic well-being. Social interventions such as counseling, advocacy, and support groups would reduce physical, psychological, and sexual violence. There must be reduced acceptance of intimate partner violence, relationship quality must be improved, and male household participation must be improved. Women must be encouraged for help-seeking behavior when in need. Community-based interventions are required to bring about gender-equitable attitudes among adolescents both boys and girls, to prevent the occurrence of intimate partner violence.

## Conclusions

More than half of the study participants have experienced controlling behavior by their partner and there is a huge impact of intimate partner violence on the mental health of women whether it is psychological, physical, or sexual. There is a need for awareness and effective management of violence against women, especially in rural areas.
